# Metastasectomy of a solitary liver tumor from prostate cancer after radical prostatectomy

**DOI:** 10.1002/iju5.12561

**Published:** 2022-12-10

**Authors:** Tomoyuki Hayashi, Kenji Yoshida, Chiharu Tamura, Yoshio Miyazawa, Masahiko Sato, Taito Miyamoto, Masayuki Nakagawa

**Affiliations:** ^1^ Department of Urology Wakakusa Daiichi Hospital Higashiosaka Osaka Japan; ^2^ Department of General Medicine Dongo Hospital Yamatotakada Nara Japan; ^3^ Department of Surgery Wakakusa Daiichi Hospital Higashiosaka Osaka Japan; ^4^ Department of Pathology Dongo Hospital Yamatotakada Nara Japan; ^5^ Sato Clinic Higashiosaka Osaka Japan; ^6^ Department of Gynecology and Obstetrics Kyoto University Graduate School of Medicine Kyoto Japan

**Keywords:** liver metastasis, metastasectomy, oligometastasis, prostate cancer

## Abstract

**Introduction:**

Metastasectomy of oligometastatic prostate cancer has the potential to contribute to improving prognosis. We report on a case of metastasectomy of solitary liver tumor after radical prostatectomy.

**Case presentation:**

An 80‐year‐old man underwent radical prostatectomy for prostate cancer, followed by radiotherapy after the operation because of increased serum prostate‐specific antigen levels of 0.529 ng/mL. Levels increased further to 0.997 ng/mL even after salvage therapy. The patient then received androgen deprivation therapy. Levels remained stable for 3 years, but rapidly increased to 19.781 ng/mL in the following 6 months. Abdominal computed tomography revealed a solitary liver tumor, and no metastasis to other sites was identified. The patient underwent liver segmentectomy. Microscopic examination of excised specimens revealed prostate cancer cells. Five years after surgery, serum prostate‐specific antigen maintained to the lowest level so far.

**Conclusion:**

Metastasectomy might be a beneficial therapeutic option to improve the prognosis for solitary metastasis from prostate cancer.

**Abbreviations & Acronyms**
ADT: androgen‐deprivation therapy
CRPC: castration‐resistant prostate cancer
CT: computed tomography
MDT: metastasis‐directed therapy
OS: overall survival
PCa: prostate cancer
PSA: prostate‐specific antigen

**Keynote message**

We report on a case of treatable liver metastatic tumor from prostate cancer. Metastatic prostate cancer is treated with androgen‐deprivation therapy in generally. This case report highlights that metastasectomy might be one beneficial option for solitary metastatic prostate cancer.

## Introduction

Metastasis of PCa commonly appears first in the lymph nodes and/or bones. Liver metastasis is found in 8.6% of CRPC. The median OS of patients with liver metastasis is 14 months, which is worse compared with patients with lymph node or bone metastasis.^1^ Treatment of metastatic PCa is usually a combination of chemotherapy, radiation, and hormonal therapy but metastasectomy is sometimes performed for oligometastatic PCa. For example, in a review of liver metastases from renal cancer, liver metastasectomy had a better prognosis than patients who did not undergo surgery, excluding poor prognostic factors such as positive resection margins, poor performance status, and the presence of lymph node metastases.^2^ While evidence for MDT for solitary liver metastases is still growing and inconclusive, it might be useful in well‐selected cases and could delay the use of systemic therapy, such as docetaxel and abiraterone.^3^ However, the recommended treatment for liver metastases of PCa is not established because of only few case reports. Here, we report a case of successful metastasectomy of a solitary liver tumor from PCa after radical prostatectomy, along with a review of the literature.

## Case presentation

**Fig. 1:**
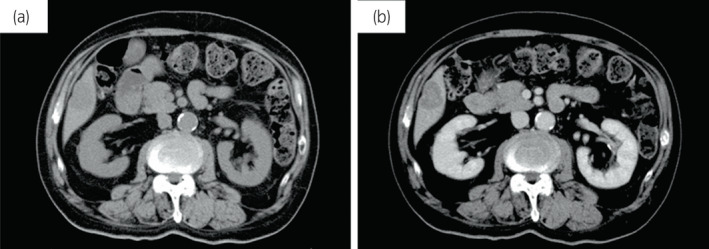
(a) Abdominal CT showed a solitary liver tumor with a diameter of 35 mm in the right posterior inferior segment of the liver. The tumor was not seen in previous scans. (b) Enhanced imaging revealed that the tumor had a concentric pattern with low density.

**Fig. 2:**
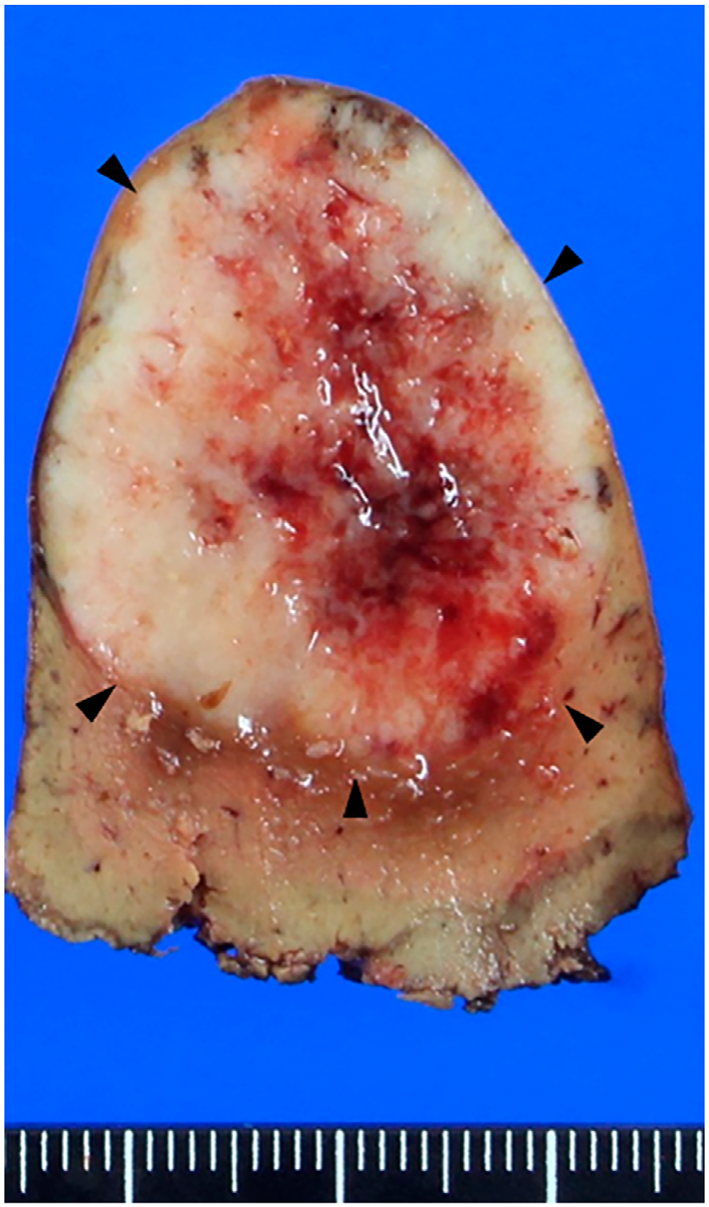
Macroscopic examination showed a solid liver tumor with central necrosis. The tumor diameter was 35 × 30 × 27 mm.

**Fig. 3:**
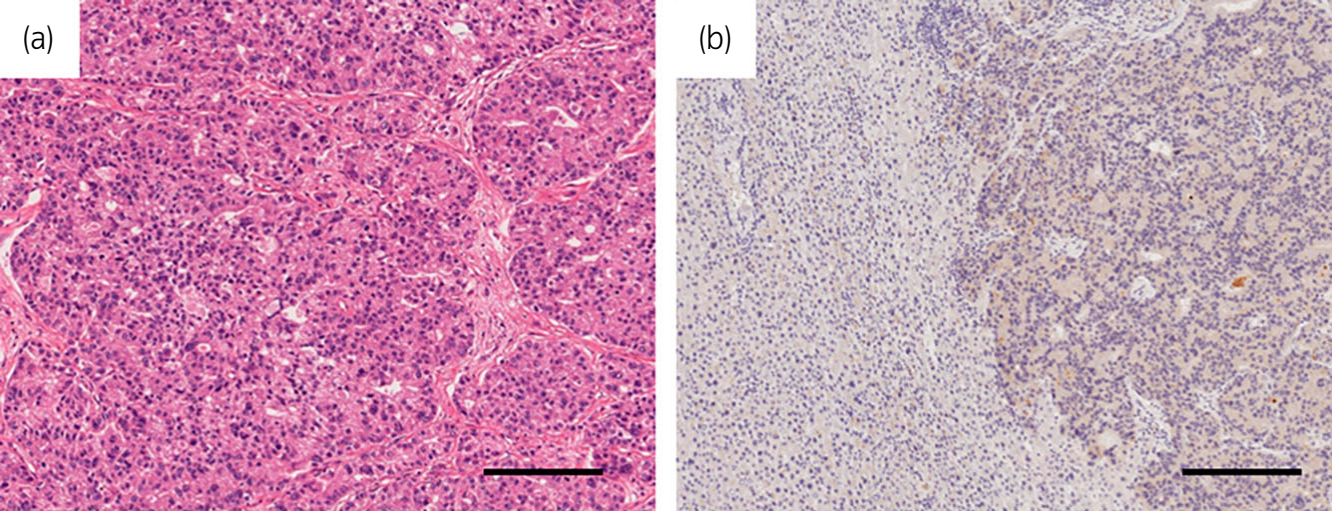
(a) Microscopic examination showed moderately differentiated adenocarcinoma with cribriform pattern, mild polygon nuclei, large nucleoli, and fine granular abundant cytoplasm. The tumor was diagnosed histologically as metastatic PCa. (b) Immunobiological staining for PSA was positive throughout the tumor cells. The scale bar represents 200 μ.

An 80‐year‐old man was diagnosed with PCa when his initial serum PSA level was 13.3 ng/mL, and he underwent radical prostatectomy (9 years previously). Microscopic findings of the prostate tissue suggested adenocarcinoma with a Gleason score of 8. During the first year of postoperative follow‐up, serum PSA gradually increased to 0.529 ng/mL. We suspected local recurrence of PCa because no obvious distant metastasis was identified elsewhere by abdominal CT and bone scintigraphy. The patient underwent 64 Gy external radiation therapy. However, serum PSA continued to rise to 0.997 ng/mL after the therapy. The patient went on to receive hormonal therapy using leuprorelin and bicalutamide/flutamide. The serum PSA remained stable for 3 years but rapidly increased to 19.781 ng/mL in the following 6 months. Abdominal CT revealed a solitary liver tumor with a diameter of 35 mm in segment 6 of the liver (Fig. 1). No metastasis to other organs was observed. Bone metastasis was not detected by ^99m^Tc bone scintigraphy. Serum alpha‐fetoprotein and protein induced by vitamin K absence or antagonist II were within the normal range. We extremely suspected the liver metastasis from PCa after consultation with liver surgeons and radiologists according to the rapidly increasing serum PSA level and the absence of obvious metastasis in other organs. Tumor biopsy was not performed due to the risk of percutaneous root dissemination.^4^ Although a definitive diagnosis could not be made preoperatively, the surgery was performed after careful explanation to the patient because of previous reports of metastasectomy for liver metastasis from PCa.^5^, ^6^ Macroscopic examination of the excised specimen revealed a solid tumor diameter of 35 × 30 × 27 mm (Fig. 2). The pathological examination showed moderately differentiated adenocarcinoma with cribriform pattern, mild polygon nuclei, large nucleoli, and fine granular abundant cytoplasm. The tumor cells showed positive immunobiological staining for PSA and cytokeratin‐19 immunobiologically (Fig. 3), which is a characteristic of PCa cells. The resected tumor was consistent with metastatic PCa, and the margin was microscopically free of cancer cell. One month after the operation, serum PSA decreased to 0.280 ng/mL. Scanning via nuclear medicine did not recognize apparent relapse. The patient received adjuvant hormonal monotherapy with leuprorelin to reduce the risk of recurrent PCa. Five years after surgery, low serum PSA <0.01 ng/mL has been achieved so far.

## Discussion

PCa metastasis commonly occurs in the lymph nodes or bones and less frequently in the liver. Pouessel *et al*. reported that 28 out of 345 patients with PCa had liver metastases, and only one had a solitary liver tumor.^7^ The autopsy study of 1589 patients by Bubendorf *et al*. indicated that hematogenous metastasis in PCa spread via three pathways: Batson's epidural venous plexus spread; cava‐type metastasis to the lungs and via the lungs to other organs; and cava‐type metastasis without lung involvement.^8^ Batson's venous plexus is an important pathway for bone metastasis from malignant tumors. The prostatic venous plexus communicates directly with Batson's epidural venous plexus and not through the vena cava. For this reason, bone metastasis from PCa is more prone to appear in the liver and lungs. Recent studies have demonstrated that liver metastasis generally occurs in conjunction with other organ metastases.^1^ Therefore, patients with liver metastases from CRPC have poorer prognosis compared with those with lymph node, bone, or lung metastases.

Oligometastatic recurrence is defined as 1–3 metastatic lesions on imaging modalities. Metastatic PCa is usually treated with ADT, although metastasectomy might be selected for oligometastatic PCa under limited circumstances. MDT such as radiation or metastases dissection is one favorable procedure for oligometastatic PCa.^9^ However, these treatment strategies are not standard because of incomplete evidence from randomized controlled trials. Battaglia *et al*. reported that, of 17 patients that had recurrent oligometastatic PCa and received metastasectomy, 16 had decreased serum PSA after the operation.^10^ The report demonstrated that it was unclear whether metastasectomy improved OS but that metastasectomy might be associated with patient benefit such as decreasing serum PSA. Only a few studies have focused on the treatment of single metastatic liver lesions from PCa.^5^, ^6^ The efficacy of metastasectomy for oligometastases to the liver is also unknown, but it might be considered as one of the treatment options.

ADT after metastasectomy is an issue that should be carefully considered. In our case, postoperative ADT might be unnecessary because of not enough efficacy of hormonal therapy before metastasectomy. On the other hand, Wang *et al*. reported a case with surgery of isolated hepatic metastasis from PCa, and the patient continued to treat Luteinizing Hormone‐Releasing Hormone agonist post‐operatively.^6^ We currently do not have standardized criteria for ADT after metastasectomy for PCa recurrence. In the present case, we comprehensively assessed ADT after metastasectomy according to the patient's age, performance status, histologic type and previous reports. It may be an option to observe without ADT in the future because of extremely low serum PSA.

In conclusion, metastasectomy in the condition of resectable tumor and good performance status might be considered as one of the beneficial therapeutic options for solitary liver metastasis from PCa.

## Author contributions

Tomoyuki Hayashi: Investigation; writing – original draft; writing – review and editing. Kenji Yoshida: Project administration; supervision. Chiharu Tamura: Conceptualization. Yoshio Miyazawa: Data curation; formal analysis. Masahiko Sato: Conceptualization. Taito Miyamoto: Conceptualization; writing – review and editing. Masayuki Nakagawa: Conceptualization.

## Conflict of interest

The authors declare no conflict of interest.

## Approval of the research protocol by an Institutional Reviewer Board

Informed consent to participate was obtained from the patient.

## Informed consent

We obtained consent from our patient for the publication of this case.

## Registry and the Registration No. of the study/trial

Not applicable.

## Ethical statement

All informed consent was obtained from the patient to publish details of his case, and his identity was protected. This report was conducted in accordance with the Declaration of Helsinki.
